# Presence of paroxysmal nocturnal hemoglobinuria in patients with idiopathic portal vein thrombosis: a single-center study

**DOI:** 10.3906/sag-1912-204

**Published:** 2020-08-26

**Authors:** Cengiz DEMiR, Senar EBİNÇ, Ömer EKİNCİ

**Affiliations:** 1 Department of Hematology, Faculty of Medicine, Yüzüncü Yıl University, Van Turkey; 2 Department of Hematology, Faculty of Medicine, Fırat University, Elazığ Turkey

**Keywords:** Portal vein thrombosis, paroxysmal nocturnal hemoglobinuria, FLAER

## Abstract

**Background/aim:**

Paroxysmal nocturnal hemoglobinuria (PNH) is a very rare clonal hematopoietic stem cell disease characterized by chronic hemolytic anemia and thrombosis. We report data from a study of the occurrence of PNH among patients with idiopathic portal vein thrombosis (PVT).

**Materials and methods:**

Patients who were followed up with the diagnosis of idiopathic PVT were enrolled into this study. Those with laboratory and/or imaging evidence of any local or systemic factor that could lead to PVT were excluded. PNH clone was examined in all patients using established FLAER methodology.

**Results:**

A total of 112 patients (42 males and 70 females), none of them had a markedly PNH clone, but 4 patients (3.6%) with confirmed tests two times had small PNH clones (size between 3.02% and 4.62%). The median ages of PNH clone (-) and PNH clone (+) patients were 42 (range; 24–59) vs 39 (range; 36–42) years, respectively. The median hemoglobin concentration, platelet count and leukocyte count were lower in the PNH clone (+) group than the PNH clone (-) group. Anemia, thrombocytopenia, and leukopenia were detected in all PNH clone (+) patients. In addition, the PNH clone positivity size in monocytes was higher than erythrocytes in all of 4 patients.

**Conclusions:**

PNH should be considered during differential diagnosis among patients with idiopathic PVT. Small PNH clones can be detected in PVT patients that we cannot clearly determine its relationship with PVT. We need furthermore studies to explore the potential role of this finding.

## 1. Introduction

Paroxysmal nocturnal hemoglobinuria (PNH) is an acquired clonal stem cell disease characterized by venous thrombosis and intravascular hemolysis, caused by a somatic mutation in the phosphatidylinositol N-acetylglucosaminyltransferase subunit A (PIGA) gene located on chromosome X [1–3]. PNH occurs with approximately equal incidence in men and women, and the overall disease prevalence has been estimated at 2–5 cases per 1,000,000. The mean age at onset of PNH is around 42 years, and the 5-year mortality rate is approximately 35% [4]. Clinically, PNH occurs in three different forms. Classic (hemolytic), bone marrow deficiency syndrome (hypoplastic), and a subclinical form [5]. Although the disease typically manifests with signs of bone marrow failure, thrombotic events, and chronic intravascular hemolytic anemia particularly those affecting the abdominal veins, are considered the most important complication during its course.

Thrombotic events are most commonly seen in the splenic, mesenteric, hepatic, renal and portal veins [6]. Additionally, PNH should be considered and screened for in cases of recurrent miscarriages and renal insufficiency are concomitant with unexplained abdominal pain, fatigue, dysphagia, dyspnea, erectile dysfunction, iron deficiency, and cytopenia accompanied by hemolysis [7,8]. Flow cytometric demonstration of absent CD59 and CD55 expression is recommended as central to the diagnosis of PNH. FLAER (Fluorescent aerolysin) assay is acknowledged as the most appropriate detection method for GPI antigens, and enables highly sensitive measurements for the detection of PNH clone sizes as low as 0.01% [9].

The aim of this study is to evaluate the presence of paroxysmal nocturnal hemoglobinuria in patients who were followed up with the diagnosis of idiopathic portal vein thrombosis in the department of hematology at our university.

## 2. Materials and methods 

### 2.1. Patients and study design

A total of 112 patients (70 females and 42 males) who were being followed up due to idiopathic PVT in our clinic were enrolled into this study. The study protocol conforms to the ethical guidelines of the 1975 declaration of Helsinki and it was approved by the ethics committee of faculty of medicine (Ethics Committee Approval date/number: 03.07.2014/04). All patients provided written informed consent for participation in the study and for publication of study data. Study procedures and data recording/handling methods were approved by the ethics committee of our university. Those with laboratory and/or imaging evidence of any systemic or local factor that could lead to PVT (including prothrombin gene mutation, factor V Leiden mutation, antiphospholipid syndrome, systemic lupus erythematosus, celiac disease, hepatic cirrhosis, chronic myeloproliferative diseases, malignancies, abdominal trauma or previous surgery) were excluded. JAK2-V617F and other activating mutations were studied to exclude chronic myeloproliferative diseases. 

### 2.2. PNH clone detection by flow cytometry for diagnosis

No specific threshold value was defined for CD55 and CD59 expression levels in PNH. It is necessary for diagnosis to show at least 2 different GPI protein deficiencies in at least two different cell lines from neutrophils, monocytes or erythrocytes by flow cytometry. However, clone size ≥1% is sufficient for diagnosis and these should be monitored for PNH symptoms. Diagnostic analyses were performed on 2 mL EDTA-blood samples. PNH clone was examined in all patients in the flow cytometry laboratory of the department of hematology at university hospital using established FLAER methodology. This method is based on the specific binding of FLAER to GPI anchors.

#### 2.2.1. Flow cytometry

**Granulocytes:** Quartet marker combinations can be used to detect PNH clones in granulocytes [10]. In this study we used FLAER, CD24, CD15, and CD45 Monoclonal antibodies against CD24, CD45 (5 μL) and CD15 (5 μL), and 5 µL of FLAER were mixed with 50 µL aliquots of EDTA blood, followed by incubation at room temperature for 15 minutes. Cellular lysis was induced by addition of 450 μL of a 1:9 dilution of BD-FACS lysing solution. Flow cytometry was conducted on a BD FACS Catto II machine, where 50.000 cells were read on the CD24 versus FLAER graphic in the FSC-SCC dot blot.

**Monocytes:** Quartet stain combinations can also be used for PNH clone detection in monocytes (10). We used FLAER, CD14, CD64, and CD45. Staining was conducted by mixing 5 μL of anti-CD14, 5 μL of anti-CD45, and 5 μL of anti-CD64 monoclonal antibodies with 5 μL of FLAER in 50 µL aliquots of EDTA blood, followed by 15 min incubation at room temperature. Lysis was conducted as for granulocyte preparations, followed by flow cytometry analysis of 5000 cells in the CD64 positive monocyte field. 

In all patients, complete blood count and biochemical parameters were measured. Complete blood count was done by the cyanmethemoglobin method in COULTER® LH 750 hematology analyzer using commercial kits. Biochemical parameters lactate dehydrogenase (LDH), aspartate transaminase (AST), alanine transaminase (ALT), bilirubins and albumin) were measured by the spectrophotometric methods in Abbot Diagnostic Architect Ci 16200 using commercial kits. The ferritine was measured by the chemiluminescence method in BİO DPC Immulite 2000 analyser using commercial kits. Portal vein thrombosis was shown by using abdominal ultrasound with doppler imaging and abdominal CT or MRI.

### 2.3. Statistical analysis

Statistical analysis of the data was performed using the IBM SPSS 22 statistical package program. Descriptive statistics for studied variables (characteristics) were presented as median, minimum and maximum values, and for categorical variables the frequency is expressed as a percentage [n (%)]. For determining the relationship between groups and categorical variables, the chi-square test was used. In order to compare PNH positive and negative patients for demographic, clinical and laboratory characteristics, the Man-Whitney U test was performed. The level of significance was set at P < 0.05. 

## 3. Results

A total of 112 patients with idiopathic PVT were enrolled into the present study. Forty two cases (37.5%) were male and 70 cases (62.5%) were female. PNH clone positivity was identified in 4 patients (3.6%), the remaining 108 patients were PNH clone negative. The median ages of PNH clone (-) and PNH clone (+) patients were 42 (range; 24–59) vs 39 (range; 36–42) years, respectively.

Anemia, thrombocytopenia, and leukopenia were detected in all of 4 (100%) PNH clone (+) patients. However, anemia for 58 (53.7%) cases, thrombocytopenia for 20 (18.5%) cases, and leukopenia for 28 (25.9%) cases were detected in the PNH clone (-) group. There was a difference between the PNH clone (-) and PNH clone (+) groups in terms of the median serum LDH level (164 U/L vs 312 U/L), total bilirubin level (12 mg/dL vs 2.6 mg/dL) and ferritin level (61.3 ng/dL vs 19.3 ng/dL). The demographic, clinical, and laboratory characteristics of these included patients are described in Table 1.

**Table 1:** Comparison for demographic, clinical, and laboratory characteristics of PNH clone positive and PNH clone negative group.

Parameters	PNH clone (-) group	PNH clone (+) group
Number of patients, n (%)	108 (96.4)	4 (3.6)
Sex, n (%)		
Male	41 (37.9)	1 (25)
Female	67 (62.1)	3 (75)
Age, year		
Median	42	39
Range	24–59	36–42
Anemia, n (%)	58 (53.7)	4 (100)
Hemoglobin level (g/dL)		
Median	10.5	10.3
Range	10.1–15.2	9.1–10.9
Leukopenia, n (%)	28 (25.9)	4 (100)
Leukocyte count (×109/L)		
Median	4.8	3.26
Range	2.1–9.8	1.8–3.3
Thrombocytopenia, n (%)	20 (18.5)	4 (100)
Platelet count (×109/L)		
Median	104.8	98.1
Range	32.2–428	87.9–105.6
Lactate dehydrogenase (U/L)		
Median	164	312
Range	108–238	284–350
Total bilirubin (mg/dL)		
Median	1.2	2.6
Range	0.7–1.6	1.9–2.8
Ferritin (ng/dL)		
Median	61.3	19.3
Range	48.8–80.8	18.1–20.8

There was 1 male and 3 female patients in the PNH clone (+) group. The clone size was between 3.02% and 4.62% in the monocytes series in these patients. PNH clone positivity size in monocytes was higher than erythrocytes in all of 4 patients. Characteristics of PNH clone (+) cases showed in Table 2.

**Table 2:** Characteristics of PNH positive cases.

Parameters	Case 1	Case 2	Case 3	Case 4
Age	36	39	42	40
Sex	Male	Female	Female	Female
Vein thrombosis area	PV	PV + SV	PV	PV
Hemoglobin (g/dL)	9.1	10.3	9.8	10.9
Leukocyte (×10^9^/L)	1.80	2.67	3.30	3.26
Platelets (×10^9^/L)	92.4	87.9	98.1	105.6
Lactate dehydrogenase (U/L)	292	284	312	350
Total bilirubin (mg/dL)	2.1	2.8	2.6	1.9
Indirect bilirubin (mg/dL)	1.2	1.7	1.8	1.3
FLAER				
Monocytes clone size (%)	3.32	3.02	4.62	3.86
Erythrocyte clone size type II (%)	0.18	0.89	0.06	1.46
Erythrocyte clone size type III (%)	1.12	2.18	0.12	0.21

PV: Portal vein; SV: Splenic vein.

## 4. Discussion

PVT may arise due to a number of factors. In the majority of cases, local factors play a role in its etiology and, frequently, more than one coexist. In the general population, the lifetime risk of developing PVT is approximately 1% [11]. PNH is very rare among patients with intra-abdominal vein thrombosis related to any cause [12,13]. In the course of PNH, venous thrombosis is the major event that affects the life. Abdominal venous thrombosis may occur as hepatic, mesenteric, splenic, renal and portal vein thrombosis [14]. Hepatic vein thrombosis is more commonly seen compared to PVT due to increased blood flow and decreased blood pressure and pH coexistent hepatic vein involvement is therefore common in patients with PVT [15,16].

During eight years of follow up in 220 patients with PNH in a previous study, pancytopenia, thrombosis and myelodysplastic syndrome were observed in 15%, 28% and 5% of patients, respectively [17]. In particular, venous thrombosis during the course of the PNH is considered a major and potentially life-limiting event as it is associated with 40%–67% of deaths among patients affected by PNH [2,13]. Although the cause of thrombosis in PNH has not been fully elucidated, it is thought that activated complement predisposes patients to thrombosis by indirectly promoting platelet aggregation and hypercoagulability. In our PNH positive cases there were no local or systemic risk factors typically associated with PVT, and there was no history of thrombosis in other intraabdominal veins (including the hepatic vein) or arterial systems. 

Intermittent hemoglobinuria, coombs-negative hemolytic anemia, LDH elevation, thrombosis, granulocytopenia and/or thrombocytopenia are often observed during the course of PNH [18]. In our PNH clone (-) group, 58 (51.8%) patients had anemia, 20 (18%) had thrombocytopenia and 28 (25%) had leukopenia. A total of 61 (54.5%) patients had splenomegaly, including all patients with leukopenia and thrombocytopenia and 13 of those with anemia. There was no evidence of hemolytic anemia in patients with splenomegaly. Therefore, it was considered that cytopenia is associated with hypersplenism in these patients. 

The PNH clone (+) patients had pancytopenia. Vitamin B12 and folic acid levels were normal in these patients with pancytopenia. Coombs tests were negative and indirect bilirubin levels were high in the patients. Retrospective analysis of their medical history identified a fluctuating course of LDH elevations. The coexistence of these findings suggested the presence of a hemolytic process. Ferritin levels of these patients were also found to be decreased, suggesting borderline iron deficiency. Spleens of these patients were also large, suggesting that eventual hypersplenism contributed to the development of cytopenia.

Data suggest that the risk of thrombosis in PNH is associated with PNH clone size [19]. According to the results of two large studies, in patients who have thrombosis, PNH clone frequency (inside granulocytes) was measured higher than 50% and 61% [19,20]. However, lower clone sizes have been detected in some reported cases of thrombosis in patients affected by PNH. Ageno et al. evaluated 202 patients with splanchnic vein thrombosis, reporting clone sizes of 0.01% and 0.16% in two patients (incidence 1%), one with PVT without any risk factors and the other with superior mesenteric vein thrombosis due to inflammatory intestinal disease, respectively [21]. Ahluwalia et al. reported PNH clones in two patients with PVT in a cohort of 142 patients with intraabdominal venous thrombosis (incidence 1.4%), and Qi et al. detected PNH clones in one patient out of 100 with PVT (incidence 1.0%) [22,23]. We excluded patients with diseases that could lead to PVT in this study, and detected a FLAER/CD24-negative PNH clones (size 3.02%–4.62%) in 4/112 patients with clinical PVT, indicating an incidence of 3.6% in this cohort. The relatively higher incidence of PNH in our cohort compared with the three other listed studies could be due to the fact that patients with known PVT related to other diseases were not included. In our study, the number of PNH clone (+) patients and the detected clone level were small. Therefore, it is not possible to say that the cause of PVT was these patients with PNH clone positivity.

It is appropriate to conduct treatment-free monitoring for asymptomatic or mildly affected patients with PNH clone sizes below 10% [24]. In our study, in the current identified cases with a PNH clone below 10% in size, we decided that anticoagulant therapy with periodic PNH clone monitoring was the most appropriate approach.

In conclusion, we report a study to assess the presence of PNH clones in patients with idiopathic portal vein thrombosis. Small PNH clones can be detected in PVT patients that not clearly determine its relationship with PVT. Because of the small number of patients in our study, it is difficult to determine both this relationship and frequency of PNH in idiopathic PVT patients. Therefore, we believe that future studies should explore the potential role of small PNH clones in this situation of patients with idiopathic PVT.

## Contribution of authors

Demir C and Ebinç S collected the data, analyzed and interpreted the data, and prepared the manuscript. Ekinci O collected the data, performed the statistical analyses and were responsible for the final editing. 

## Informed consent

Written informed consent was obtained from the patients who participated in this study.
